# Heterologous expression and biochemical characterization of a GHF9 endoglucanase from the termite *Reticulitermes speratus* in *Pichia pastoris*

**DOI:** 10.1186/s12896-018-0432-3

**Published:** 2018-06-01

**Authors:** Pengfei Zhang, Xianghua Yuan, Yuguang Du, Jian-Jun Li

**Affiliations:** 1 Sichuan Normal University, College of Life Science, Chengdu, 610101 China; 2 National Key Laboratory of Biochemical Engineering, National Engineering Research Center for Biotechnology (Beijing), Key Laboratory of Biopharmaceutical Production & Formulation Engineering, PLA, Institute of Process Engineering, Chinese Academy of Sciences, No. 1 North 2nd Street, Beijing, 100190 China

**Keywords:** *Reticulitermes speratus*, GHF9 endoglucanse, Heterologous expression, *Pichia pastoris*, Enzymology

## Abstract

**Background:**

Cellulases are of great significance for full utilization of lignocellulosic biomass. Termites have an efficient ability to degrade cellulose. Heterologous production of the termite-origin cellulases is the first step to realize their industrial applications. The use of *P. pastoris* for the expression of recombinant proteins has become popular. The endoglucanase from *Reticulitermes speratus* (RsEG)*,* belonging to glycoside hydrolase family 9 (GHF9), has not been produced in *P. pastoris* yet.

**Results:**

A mutant RsEG_m_ (G91A/Y97W/K429A) was successfully overexpressed in *P. pastoris*. RsEG_m_, with optimum pH 5.0, was active over the pH range of 4.0 to 9.0, and exhibited superior pH stability over between pH 4.0 and pH 11.0. It displayed the highest activity and good stability at 40 °C, but lost activity quickly at 50 °C. The apparent kinetic parameters of RsEG_m_ against Carboxymethyl Cellulose (CMC) were determined, with *K*_*m*_ and *V*_*max*_ of 7.6 mg/ml and 5.4 μmol/min•mg respectively. Co^2+^, Mn^2+^ and Fe^2+^ enhanced the activity of RsEG_m_ by 32.0, 19.5 and 11.2% respectively, while Pb^2+^ and Cu^2+^ decreased its activity by 19.6 and 12.7% separately.

**Conclusions:**

RsEG_m_ could be overexpressed in *P. pastoris*. It was stable between pH 4.0 and pH 11.0, and exhibited higher stability at temperatures ≤ 40 °C. This endoglucanase may have potential to be used in the field of laundry, textile and lignocellulose-based biofuels and chemicals.

**Electronic supplementary material:**

The online version of this article (10.1186/s12896-018-0432-3) contains supplementary material, which is available to authorized users.

## Background

Lignocellulosic biomass obtained as agricultural and industrial byproducts is an abundant, inexpensive and renewable source, and is a desirable feedstock for the sustainable production of liquid fuels and chemicals through the biorefinery processes [1, 2]. Lignocellulose is mainly composed of cellulose, hemicellulose and lignin, among which cellulose is the major polysaccharide. The turnover of cellulose plays an important role in global carbon cycle for all living organisms. In nature, the degradation of cellulose is mainly performed by cellulases produced by microorganisms. To breakdown cellulose efficiently, three classes of cellulases are needed to work synergistically: endoglucanases (EGLs, EC 3.2.1.4), cellobiohydrolases (CBHs, EC 3.2.1.91) and β-glucosidases (BGLs, EC 3.2.1.21) [3, 4]. EGLs hydrolyze intramolecular β-1,4-glucosidic linkages in cellulose randomly, whereas CBHs cleave cellulose from the reducing and non-reducing ends in a progressive process. BGLs degrade cellobiose into glucose. According to the CAZy (Carbohydrate-Active enZYmes) database, where glycosidases are classified according to similarities in the protein sequence and three-dimensional structure, cellulases belong to glycoside hydrolase families (GHF) 5 to 10, 12, 26, 44, 45, 48, 51, 61 and 74, etc. [5].

Termites (Isoptera or Termitoidae) are the main degraders in tropical and subtropical regions. They have a profoundly efficient ability to degrade cellulose [6–8], and can digest 74 to 99% of the cellulose present in the plant material they ingest [9]. Thus termite guts are regarded as ‘the world smallest bioreactor’ [10, 11]. Termites are classified into higher- and lower ones based on the presence or absence of flagellated protistan symbionts in their hindguts [7, 8]. Many studies have been performed in lower termites. This group of termites contains a dual cellulose-degradation mechanism: endogenous cellulases and symbiotic cellulases degrade cellulose cooperatively [7, 8, 12, 13]. All endogenous EGLs exclusively belong to the glycoside hydrolase family (GHF) 9 [7–9, 13–15]. Cellulases of flagellate origin have also been identified as members of GHF5, GHF7 and GHF45 from hindgut flagellates of *Coptotermes formosanus*, *C. lacteus*, *Mastotermes darwiniensis* and *Reticulitermes speratus* [7]. CBHs are only found in the hindgut of lower termites [7, 16], whereas both EGLs and BGLs are found in the midgut and hindgut of lower termites [11]. In comparison, higher termites, which do not have flagellates in their hindguts, account for over 75% of termite species. The cellulolytic systems of higher termites are different from those of lower ones. Studies have demonstrated that the majority of cellulase activity of higher termites takes place in the midgut, suggesting that they mainly depend on endogenous cellulases for cellulose degradation [6, 7, 15]. Metagenomic analysis revealed a diverse set of genes related to glycoside hydrolases in the hindgut of a higher termite *Nasutitermes* sp., implying that hindgut microbes also play an important role for cellulose degradation [17]. Proteome analysis of the bacterial community in a higher termite *Nasutitermes corniger* indicated that bacterial enzymes play more significant roles in metabolism than in activities related to cellulose degradation [18].

Since identification of an endogenous cellulase gene (*RsEG*) in *Reticulitermes speratus* by Watanabe et al. [14], encoding an EGL in GHF9, a lot of insect-origin GHF9 cellulase genes have been cloned and/or analyzed, such as *NtEG* from the higher termite *Nasutitermes takasagoensis* [19], *NwEG* from *Nasutitermes walker* [19], *TeEG-I* from the cricket *Teleogryllus emma* [20], *CfEG3a* and *CfEG5* from Formosan subterranean termite (*Coptotermes formosanus*) [21, 22], *Cell-1* from *Reticulitermes flavipes* [23], *TcEG1* from red flour beetle *Tribolium castaneum* [24, 25], *CgEG1* from the Brazaian termite *Coptotermes gestroi* [26], *MbEG1* from the fungus-growing higher termite *Macrotermes barneyi* [27], etc.. These cellulase-coding genes are predominantly expressed in salivary glands/midgut/foregut [7, 8, 15, 23]. Salivary gland−/midgut-secreted endogenous cellulases may work in both the midgut and the hindgut while symbiont cellulases work only in the hindgut [7, 8, 15]. The roles of the endogenous cellulases played in the gut system are postulated to be as important as those their symbionts produced (mostly GHF5, GHF7 and GHF45) in the hindgut for converting cellulose termites ingest [7, 8, 12].

Heterologous expression of the insect-origin cellulase genes is the first step to realize their industrial applications. Some insect GHF9 endoglucanases have been heterologously overexpressed and biochemically characterized. For instance, RsEG was successfully overexpressed in *E. coli* by using a directed evolution approach, and the obtained mutant A18 was not only efficiently expressed in *E. coli*, but also showed a 20-fold higher activity than native RsEG [28, 29]. Later, active RsEG and NtEG were also successfully obtained in *Aspergillus oryzae* [30]. Recombinant CfEG3a, Cell-1, CfEG5a, CgEG1 and MbEG1 were also produced in *E. coli*, and could hydrolyze cellulose [21–23, 26, 27]. Moreover, active TeEG-I (baculovirus-infected insect Sf9 cells) [20], Cell-1 (baculovirus-infected insect Sf9 cells) [23], TcEG1 (Drosophila S2 cells and *S. cerevisiae*) [24, 25], and MbEG1 (*P. pastoris*) were successfully overexpressed in the eukaryotic expression systems [27]. So far, only the kinetic parameters of several insect-origin GHF9 endoglucanases were determined, including TeEG-I, CfEG3a, RsEG, NtEG, Cell-1, CfEG5 and CgEG1 [20–23, 26, 30]. Eight biochemically characterized insect GHF9 endocellulases including RsEG, NtEG, CfEG3a, CfEG5a, TcEG1, MbEG1, Cell-1 and TeEG-I were aligned, and the sequence identity between them is from 61.6 to 63.2% (Additional file 1).

*Reticulitermes speratus* is one of the most-extensively investigated termites in terms of its cellulolytic systems [7, 8, 14]. Glycoside hydrolases from *Reticulitermes speratus*, belonging to GHF3, GHF7, GHF9 and GHF45, were heterologously overexpressed in *E. coli*, *S. crevisiae* and *A. oryzae* respectively [31, 32]. However, no glycosidases from *Reticulitermes speratus* have been produced in *P. pastoris*. So far, only one GHF9 endo-glucanase from termite *Macrotermes barneyi* was successfully expressed in *P. pastoris*. Methylotrophic yeast *P. pastoris* can strongly over-expresses foreign proteins and serve as an expression system for insect proteins [33, 34]. Therefore, in this study, an RsEG mutant (G91A/Y97W/K429A) named as RsEG_m_ was heterologously overexproduced in *P. pastoris*, and recombined RsEGm was fully characterized, including optimal pH and temperature, pH and thermal stability, kinetic parameters and impact of divalent metal ions on the enzymatic activity.

## Results

### Overexpression of RsEG_m_ in *P. pastoris*

**Fig. 1:**
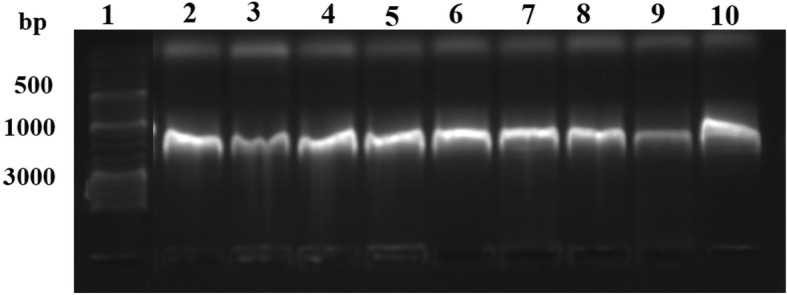
Agarose gel for PCR products of nine randomly picked transformants. Lane 1: DNA ladder; Lanes 2-10: PCR products of 9 randomly picked transformants (pJL36A - pJL36I)

The codon optimized gene *RsEG*_*m*_ encoding endoglucanase from *Reticulitermes speratus* (GenBank: AB008778.2) with three mutations (G91A/Y97W/K429A) was synthesized [29] (Additional file 2), and cloned into the expression vector pPICZαA at the restriction sites of *EcoR*I and *Xba*I. The obtained construct pJL36 was confirmed by DNA sequencing. Linearized construct by *Bst*XI was transformed into *P. pastoris* GS115 by electroporation. Nine transformants were randomly picked, grown and used for PCR. The agarose gel electrophoresis results of the PCR products of nine transformants, corresponding to the size (~1800 bp) of *RsEG*_*m*_ plus α-factor signal sequence, *c-myc* epitope and His_6_ tag, confirmed that the *RsEG* gene was successfully inserted into genome *P. pastoris* GS115 (Fig. 1).

Five (pJL36A, pJL36C, pJL36E, pJL36G and pJL36I) out of nine transformants were used to screen the one with the highest protein expression level. As shown in Additional file 3, pJL36A, pJL36C and pJL36E showed higher protein production level (Additional file 1: Figure S1). According to the amino acid sequence of pJL36 and the C-terminal peptide containing c-myc epitope and His_6_ tag, the predicted molecular weight of pJL36 is around 48.7 kDa, which was much lower than the apparent molecular weight on SDS-PAGE (Additional file 3). The difference between the predicted molecular weight and the apparent one for pJL36 was possibly due to the fact that highly glycosylated proteins are usually obtained when they are overexpressed in *P. pastoris* [32]. After pJL36A, pJl36C and pJL36E were further induced with 0.5% methanol for different time intervals, it was found pJL36C produced higher protein yield and used for subsequent large-scale overexpression (Additional file 4). Overexpression of pJL36C was also confirmed by western blotting (Additional file 5) and CMC activity.

### Determination of pH optima and pH stability of RsEG_m_

Britton-Robinson (B & R) buffer is a “universal” buffer used for the range of pH 3.0 to pH 11.0, so it was chosen for determining optimal pH and pH stability of RsEG_m_.

**Fig. 2:**
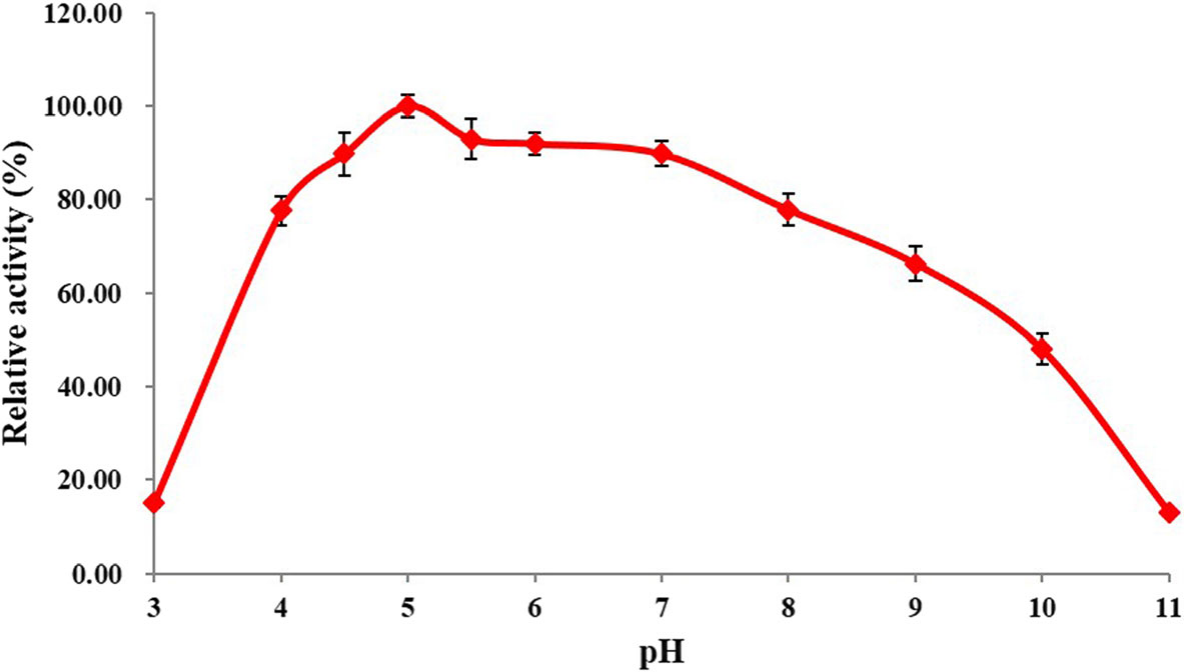
Effects of pH on RsEG_m_ activity. Values are expressed as the means of three replicates ± standard deviation. The activity of the enzyme at pH 5.0 and 37 °C (6.7 U/mg) was defined as 100%

As shown in Fig. 2, RsEG_m_ showed the highest activity at pH 5.0, and retained original activities above 77.6% between pH 4.0 and pH 8.0. It maintained 66.3 and 48.0% residual activities at pH 9.0 and pH 10.0 respectively, and exhibited very low activity (< 15.0% residual activities) at pH 3.0 and pH 11.0. Therefore, RsEG_m_ was active over the pH range of 4.0 to 9.0. Our result is slightly different from the published one for recombinant WT RsEG produced in *A. oryzae*: it had similar optimal pH (pH 5.5) to RsEG_m_, but exhibited superior activity within a narrow pH range (pH 5.0 - pH 7.5) [30].

**Fig. 3:**
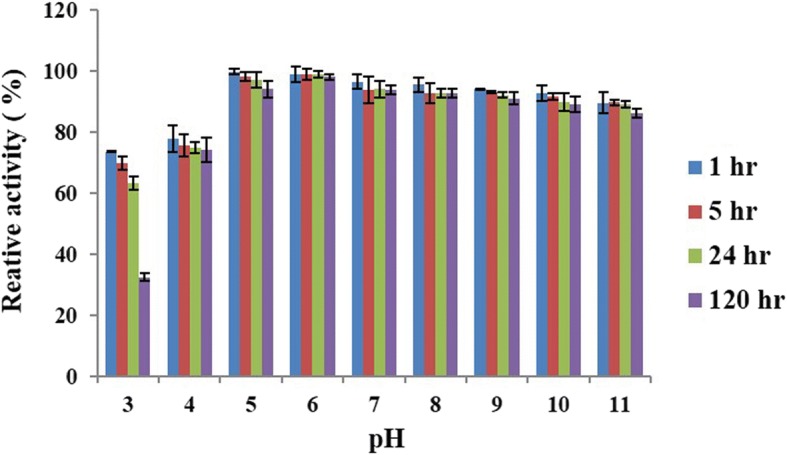
pH stability of RsEG_m_. The pH stability assay was investigated by first pre-incubating RsEG_m_ in 50 mM B & R buffer at different pH values (pH 4.0 to 11.0) at 4°C for 1 h, 5 h, 24 h, and 120 h respectively. The residual activities were then measured immediately under standard conditions (optimal pH, 37°C for 15 min). The initial activity at optimal pH (5.0) and 37°C (6.7 U/mg) was taken as 100%, and the percentage of the residual activity at different time points and pH values against the original one at optimal pH (5.0) was calculated. Values are expressed as the means of three replicates ± standard deviation

The pH stability of RsEG_m_ was investigated after being pre-incubated for a fixed time over the pH range of 3.0 to 11.0 (Fig. 3). Notably, RsEG_m_ was stable over the pH range of 4.0 to 11.0, retaining more than 74.0% of original activity after 120 h. It was unstable at pH 3.0, and only kept 32.5% of initial activity after 120 h. RsEG_m_ exhibited good pH stability over a wide pH range (pH 4.0 - pH 11.0). The results are very similar to the reported ones for recombinant WT RsEG in *A. oryzae*, in which it retained over 80% initial activity after 20 h of pre-incubation between pH 5.0 and 10.0, and lost activity sharply at pH 3.0 [30].

### Determination of optimal temperature and thermal stability of RsEG_m_

**Fig. 4:**
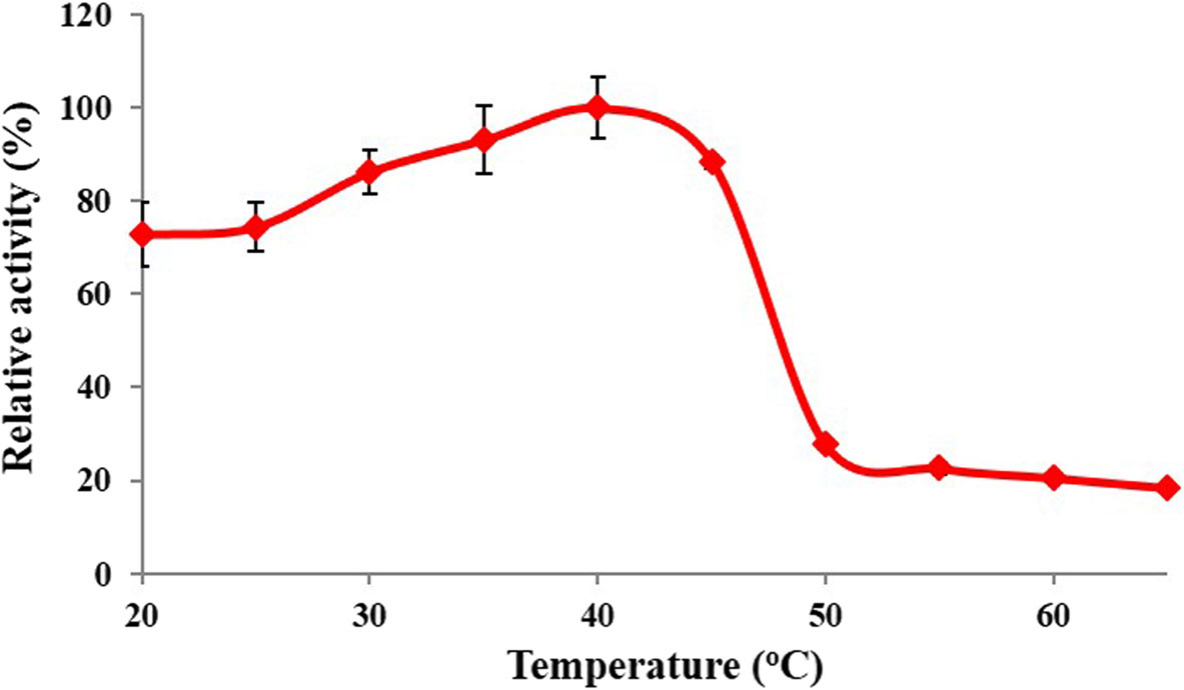
Effects of temperature on RsEG_m_ activity. Values are expressed as the means of three replicates ± standard deviation. The original activity at pH 5.0 and 40 °C was taken as 100% (7.9 U/mg)

The optimal temperature of RsEG_m_ was determined (Fig. 4). It showed the highest activity at 40 °C, and kept > 72.8% of residual activity between 20 and 45 °C. However, it lost activity rapidly when temperature rose up to 50°C, retaining only 27.7% of original activity. It maintained only 18.4% of residual activity at 65°C. Our result is very similar to the published one for recombinant WT RsEG in *A. oryzae*, where it had optimal activity at 45°C, and lost activity sharply at temperatures above 50°C [30].

**Fig. 5:**
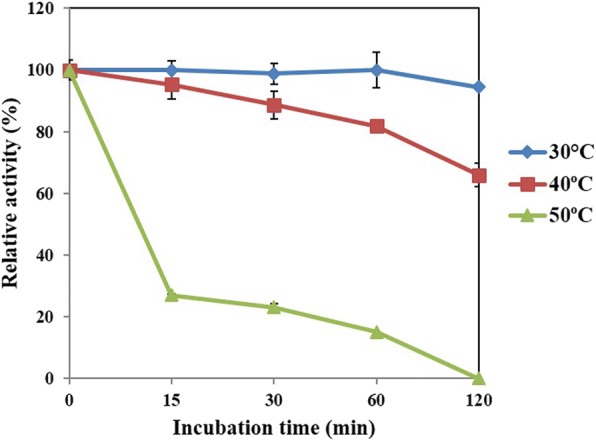
Thermal stability of RsEG_m_. RsEG_m_ was pre-incubated for varied times (15 min to 2 h) at pH 5.0, and 30°C, 40°C and 50°C respectively, and samples were chilled on ice for at least 10 min. Afterwards the residual activities were measured under standard conditions (optimal pH, 37°C for 15 min). The original activity at pH 5.0 and 37°C (6.7 U/mg) was taken as 100%, and the percentage of the residual activity at different time points and temperatures against the initial one was calculated. Values are expressed as the means of three replicates ± standard deviation

The thermal stability of RsEG_m_ was investigated after being pre-incubated for a fixed time at pH 5.0, and at 30, 40 and 50 °C respectively (Fig. 5). RsEG_m_ was stable at 30°C, and lost only 5.5% of original activity after 120 h of pre-incubation. It kept 66.0% of initial activity at 40°C after 120 h. It was completely inactivated at 50 °C after 120 h, and retained only 15.1% of original activity at 50°C after 1 h. Therefore, RsEG was a thermolabile endocellulase, and was fairly stable below 30°C. The results are very similar to the reported ones for recombinant WT RsEG in *A. oryzae*, where it retained over 80% of maximum activity after 30 min-incubation at temperatures as low as 45°C and was not stable at temperatures above 50°C [30].

### Determination of kinetic parameters of RsEG_m_

**Fig. 6:**
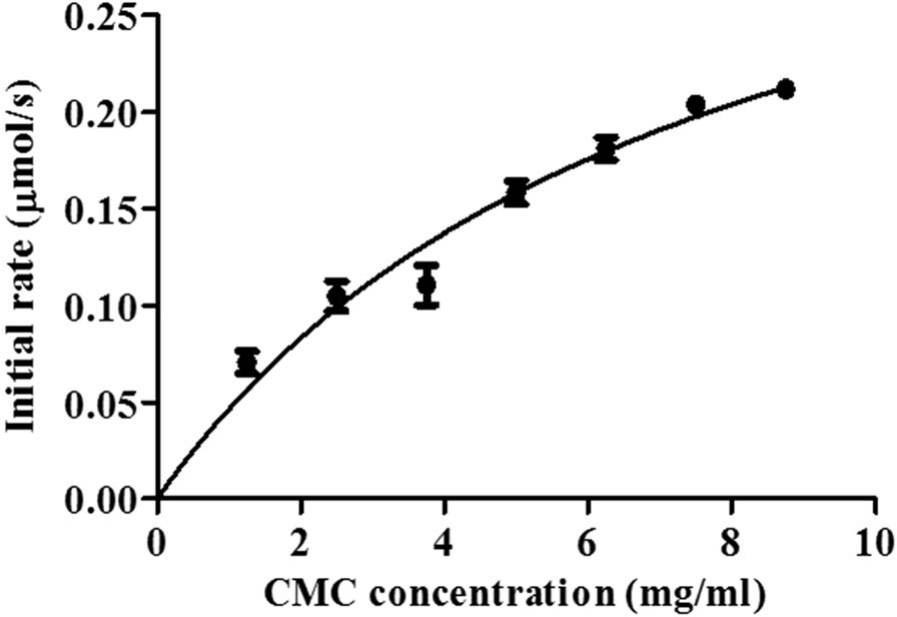
Effects of CMC concentration on RsEG_m_ activity. Values are expressed as the means of three replicates ± standard deviation

The kinetic parameters of recombinant RsEG_m_ against CMC were determined by substrate hydrolysis from 0.2 to 2% (*w*/*v*) for 5 min. The deduced kinetic values were apparent parameters since saturation was not achieved even when high CMC concentrations were used (Fig. 6). The apparent *K*_*m*_ and *V*_*max*_ values of RsEG towards CMC were 7.6 mg/ml and 5.4 μmol/min•mg respectively.

### Effects of divalent metal ions on enzyme activit*y*

**Fig. 7:**
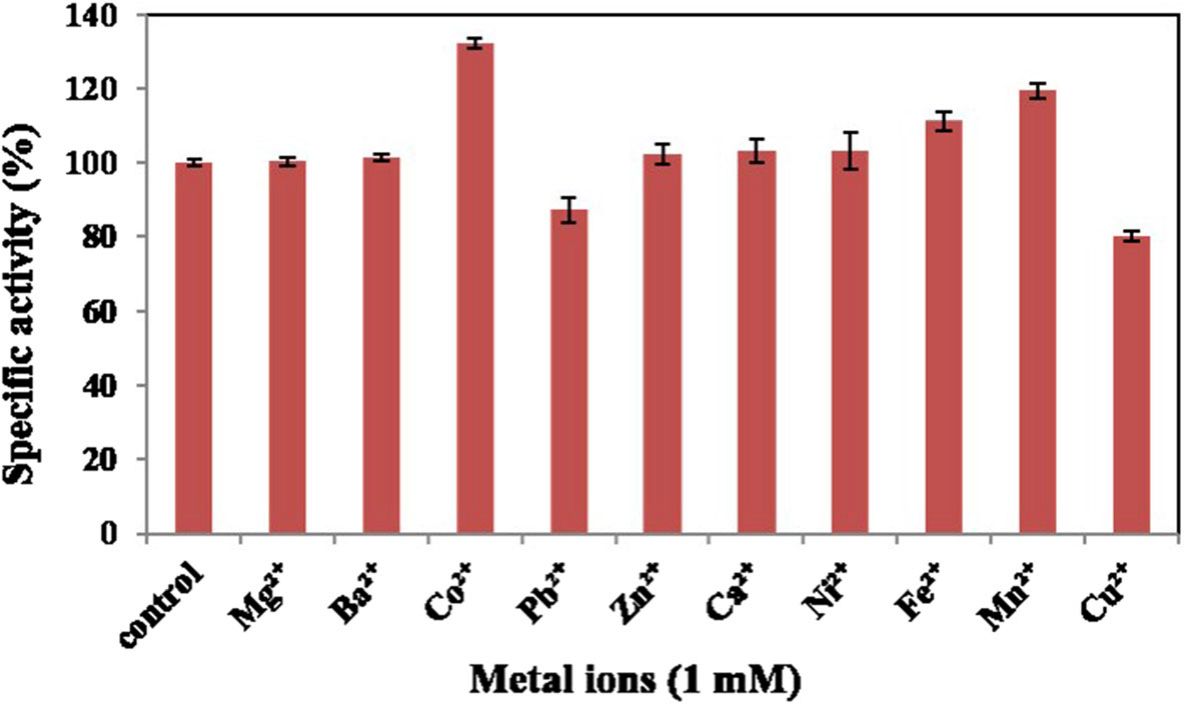
Effects of divalent metal ions on RsEG_m_ activity. The effects of metal ions on the catalytic activity of RsEG_m_ were determined by adding 1 mM of various divalent metals to the standard enzyme assay system (pH 5.0, 37°C for 15 min). The activity at pH 5.0 and 37°C (6.7 U/mg) in the absence of divalent metal ions (control) was taken as 100%, and the percentage of the activity in the presence of different divalent metal ions against the control was calculated. Values are expressed as the means of three replicates ± standard deviation

The effects of divalent metal ions on RsEG_m_ activity were examined (Fig. 7). Co^2+^, Mn^2+^ and Fe^2+^ upregulated the cellulolytic activity of RsEG_m_ by 32.0, 19.5 and 11.2% respectively, while Pb^2+^ and Cu^2+^ decreased the activity of RsEG_m_ by 19.6 and 12.7% separately. Other divalent metal ions didn’t show obvious influence on the catalytic activity of RsEG_m_.

## Discussion

In the present study, a GHF9 endoglucanase RsEG mutant (RsEG_m_) from *Reticulitermes speratus* was overexpressed in *P. pastoris* and characterized. BLAST (Basic Local Alignment Search Tool) search of RsEG resulted in significant matches of GHF9 cellulases originating from insects. Among top 100 Blast Hits, only nine endo-cellulaseas were heterologously overexpressed and partly characterized. As far as we know, this is the first report on heterologous expression of active RsEG in *P. pastoris.* Until now, only one insect endo-cellulase belonging to GHF9, MbEG, was heterologously overexpressed in *P. pastoris* [27]. Though active WT RsEG was not produced in *E. coli* and *S. cerevisiae*, an active mutant A18 was successfully obtained through DNA shuffling of four orthologous parental cDNAs in *E. coli* [28]. Active recombinant RsEG was also successfully overexpressed in *A. oryzae* [30]. So far, most insect GHF9 endoglucanases were produced in *E. coli* [21–23, 26, 27], and several were even expressed in the complicated baculovirus expression system [23, 24].

In comparison with other insect GHF9 endo-cellulaseas, RsEG_m_ exhibited higher specific activity than Cell-1 (~1 U/mg) and comparable activity to crude CfEG3a (14-19 U/mg) [21, 23], and much lower specific activity than other characterized insect GHF9 endoglucanases such as recombinant RsEG in *A. oryzae* (112 U/mg), CfEG5 (325 U/mg), NtEG (105 U/mg), MbEG1 (223.9 U/mg) and TeEG1 (948.1 U/mg) [20, 22, 27, 30]. The lower activity of RsEG_m_ recombined in *P. pastoris* could be presumably due to the reason that RsEG_m_ produced in *P. pastoris* was not correctly folded and/or glycosylated.

It appears that most termite GHF9 endoglucanases had optimal pH value around 5.0-6.0, including CfEG3a from *Coptotermes formosanus* (pH 5.0) [21], MbEG1 from *Macrotermes barneyi* (pH 5.5) [27], CfEG5 from *Coptotermes formosanus* (pH 5.6) [22], NtEG from *Nasutitermes takasagoensis* (pH 6.0) [30], and CgEG1 from *Coptotermes gestroi* (pH 6.0) [26]. The optimum pH of TeEG-I from the cricket *Teleogryllus emma* with 61% sequence identity to RsEG*,* was determined to be 5.0 [20]. By contrast, recombinant TcEG1 from red flour beetle *Tribolium castaneum* showed the maximum activity at pH 12.0. Only one neutral insect-origin GHF9 endocellulase – Cell-11, from the termite *Reticulitermes flavipes*, was identified and characterized, with optimal pH between 6.5 and 7.5 [23]. Therefore, most biochemically characterized insect GHF9 endoglucanases were classified as acidic cellulases. Comparison of the characterized insect GHF9 endoglucanases revealed that most of them were active over a wide pH range, including RsEG_m_ (pH 4.0 - pH 9.0), CfEG5 (pH 3.6 - pH 9.6), TeEG-I (pH 3.0 - pH 8.5) and Cell-1 (pH 4.0 - pH 9.0) [20, 22, 23]. In contrast, MbEG1 (pH 4.5 - pH 6.5), TcEG1 (pH 8.0 - pH 12.0), and RsEG produced in *A. oryzae* showed higher activity over a narrow pH range (pH 5.0 - pH 7.5) [27, 30]. Moreover, RsEG_m_ exhibited higher thermal stability than recombinant WT NtEG in *A. oryzae* and MbEG, which retained over 75% of maximal activity after 30 min of incubation at pH 3.5 to pH 8.0 and was stable after 20 h of pre-incubation between pH 5.5 and pH 9.0 respectively [27, 30].

Just like RsEG_m_, TeEG-I, CfEG3a, CfEG5, MbEG1 and recombinant TcEG1 had highest enzymatic activity at around 40°C [20–22, 25, 27]. The optimal temperatures of CgEG1, and Cell1 were 50°C and in the range of 50-60°C respectively [23, 26]. Recombinant NtEG in *A. oryzae* had optimal activity at 65°C [30]. Until now, only the thermal stabilities of several insect GHF9 endoglucanases were investigated. For example, MbEG1 retained 60% of its maximum activity after 30 min of pre-incubation at 50°C, and dramatically lost activity at temperatures above 55°C [27]. The activity TeEG-I was stable up to 45°C during 10 min of incubation, but was lost after 10 min-incubation at 75°C [20]. The t_1/2_ values of CgEG1 at 45, 50, 60 and 70 °C were 46.21, 8.2, 1.48 and 0.35 min, respectively [26]. NtEG retained 60% of maximum activity after 30 min of pre-incubation at temperatures as low as 60°C, and lost activity sharply above 60°C [30]. Thus, it seems that NtEG and MbEG1 were thermally stable than RsEG, whereas it displayed comparable thermal stability to TeEG-I and CgEG1 [20, 26, 27, 30].

In the case of the kinetic parameters of insect GHF9 endocellulases, the apparent *K*_*m*_ value of RsEG_m_ is comparable to that of Cell-1 expressed by BEVS (9.9 mg/ml) and in *E. coli* (14.7 mg/ml) [23], and is much higher than those of WT RsEG expressed in *A. oryzae* (2.0 mg/ml), CfEG3a (2.2 mg/ml), NtEG (4.7 mg/ml), and CfEG5 (5.6 mg/ml) [21, 22, 30]. Therefore, it showed lower binding affinity towards CMC. Although its *V*_*max*_ value was much higher than those of Cell-1 produced by BEVS (1.06 μmol/min•mg) and in *E. coli* (0.84 μmol/min•mg) [23], it was greatly lower than those of WT RsEG expressed in *A. oryzae* (1429 U/mg) [30], CfEG3a (590 U/mg) [21], NtEG (1667 U/mg) [30], and CfEG5 (548 U/mg) [22].

A lot of studies towards impact of divalent metal ions on endo-cellulases were done [35]. However, until now, there was only one report on insect-origin GHF9 endoglucanases that Ca^2+^ slightly enhanced CMC activity of Cell-1, and stabilized its activity over time [23]. In our study, Ca^2+^ didn’t show obvious impact on RsEG_m_. Therefore, it seems that same metal ions had different influence on GHF9 endoglucanases from different species of insects.

## Conclusions

In summary*,* a GHF9 endoglucanase RsEG mutant (RsEG_m_) from *Reticulitermes speratus* was successfully recombined in *P. pastoris* and characterized in detail. Recombinant RsEG_m_ showed the highest activity at pH 5.0 and 40 °C, and was very stable between pH 4.0 and pH 11.0. It exhibited higher stability at temperatures ≤ 40°C and was unstable above 45°C. The apparent *K*_*m*_ and *V*_*max*_ values of RsEG_m_ against CMC were 7.6 mg/ml and 5.4 μmol/min•mg respectively. Co^2+^, Mn^2+^ and Fe^2+^showed some stimulatory effects on RsEG_m_, while RsEG_m_ was inhibited by Pb^2+^ and Cu^2+^. Therefore, RsEG_m_ exhibited good pH and thermal stability to an extent, and activities of insect endoglucanases may be enhanced by some metal ions.

## Methods

### Materials

Chemicals were from Sigma, Merck or Ameresco. Oligonucleotides and the codon-optimized *RsEG*_*m*_ gene encoding an endo-β-1,4-glucanase from the termite *Reticulitermes speratus* with three mutations (G91A/Y97W/K429A) were synthesized by Shanghai Sangon Biotech Co. Ltd. (China) (The codon-optimized gene sequence was provided in Additional file 2). All restriction endonucleases were from Fermentas (Pittsburgh, Pennsylvania, USA) or Takara Biotechnology (Otsu, Shiga, Japan). The kits used for molecular cloning were from Omega Bio-tek (Norcross, Georgia, USA) or Takara Biotechnology. The expression vector pPICZαA was from Invitrogen. Super GelRed was purchased from US Everbright Inc.. Antibodies and chemical reagents used for Western blot were from Tiangen (China).

### Bacterial strains, plasmids, and media

*E. coli* DH5α was used for routine DNA transformation and plasmid isolation. *P. pastoris* GS115 was utilized for cellulase overexpression. *E. coli* DH5α was routinely grown in Luria-Bertani broth at 37°C with aeration or on LB supplemented with 1.5% (*w*/*v*) agar. 25 μg Zeocin/ml was added when required. *P. pastoris* GS115 was routinely grown in YPD (Yeast Extract Peptone Dextrose Medium) at 30°C with aeration or on YPD supplemented with 1.5% (w/v) agar. For RsEG_m_ overexpression, *P. pastoris* was first grown overnight in BMGY (buffered complex glycerol medium), then in baffled flasks in BMMY (buffered complex methanol medium) for a couple of days. YPD, BMGY and BMMY were prepared according to the standard protocol.

### DNA manipulations

General molecular biology techniques were carried out by standard procedures [36]. Restriction and modification enzymes were used following the recommendations of the manufacturers. DNA fragments were purified from agarose gels using the DNA gel extraction kit. Plasmid DNA was isolated using the plasmid miniprep kit.

The plasmid for the synthesized *RsEG*_*m*_ gene with the restriction sites of *EcoR*I and *Xba*I at 5′ and 3′-terminal respectively, which was cloned into PUC57, was digested with restriction enzymes *EcoR*I and *Xba*I, and re-cloned into the vector pPICZαA digested with *EcoR*I and *Xba*I, respectively. The final construct was confirmed by DNA sequencing, and named as pJL36.

### Screening of recombinant colonies by direct PCR and expression

The construct pJL36 was linearized with *Bst*XI and transformed into *P. pastoris* GS115 by electroporation following the manufacturer’s recommendation. Zeocin-resistant *P. pastoris* clones were grown on YPDS plates containing 100 μg/ml Zeocin. 10-20 colonies were picked and streaked for single colonies on fresh YPD or YPDS plates containing 100 μg/ml of Zeocin.

Nine transformants were randomly selected and grown overnight in YPD. 10 μl of a *Pichia pastoris* culture was placed into a 1.5 ml microcentrifuge tube, and 1 μl of the culture was diluted with 9 μl water. Then 5 μl of a 5 U/μl solution of lyticase was added and incubated at 30°C for 10 min. The sample was frozen at − 80°C for 10 min. A 50 μl PCR was set up using *Taq* polymerase, 5′ *AOX1* primer and 3′ *AOX1* primer. A 10 μl aliquot was analyze by agarose gel (1%) electrophoresis.

The above nine transformants were screened for protein expression by a small-scale protein expression following the manufacturer’s protocol. The supernatants were precipitated with cold acetone, and the precipitated samples were used for SDS-PAGE (12% polyacrylamide gels) analysis.

Five (pJL36A, pJL36C, pJL36E, pJL36G, and pJL36I) out of nine transformants with the higher cellulase expression level were further screened and optimized (induced for different time intervals). 0.5% Methanol was added every 24 h until the optimal induction time is reached. The crude protein samples were analyzed by SDS-PAGE as above.

### Protein overexpression

The transformant showing the highest protein expression level was used for large-scale expression (100 ml) in baffled flasks according to the standard protocol. Protein expression was induced with 0.5% methanol for 4 days. The supernatants were harvested by centrifugation at 5000 *g* and 4°C for 15 min, and precipitated with 80% (NH_4_)_2_SO_4_. The precipitated proteins were redissolved in buffer A (50 mM Tris-HCl, pH 8.0, 0.5 M NaCl), and stored at 4 °C. The enzyme purity was analyzed via SDS-PAGE. The protein concentration was determined by the Bradford method using bovine serum albumin as a standard. For Western blot, proteins were transferred from the gel onto a PVDF membrane. The membrane was blocked with 5% (*w*/*v*) skimmed milk in TBST (20 mM Tris/HCl, pH 7.5, 150 mM NaCl, 0.05% Tween 20), incubated with the murine monoclonal anti-polyhistidine immunoglobulin G (IgG), rinsed three times with TBST, incubated with the goat anti-mouse IgG conjugated with alkaline phosphatase, rinsed three times with TBST, and detected with BCIP (5-bromo-4-chloro-3-indolyl phosphate)/NBT (nitro blue tetrazolium) solution.

### Enzyme activity assay

All enzymatic assays were carried out in triplicate. Cellulase activity was assayed by measuring the amount of reducing sugars released from CMC (Carboxymethyl Cellulose) using the DNS (3,5-dinitrosalicylic acid) method [37]. D-Glucose was used as a standard. The standard assay mixture (1 mL) consisted of 0.5% CMC (w/v) and appropriately diluted enzyme solution in 50 mM B & R (Britton and Robinson) buffer (pH 5.5), and enzymatic reactions were performed at 37°C for 15 min. Reactions were stopped by adding 1.5 ml DNS reagent, followed by boiling for 5 min, then cooled down by running tap water. Finally, 2.5 ml of deionized water was added, and the absorbance at 540 nm was measured. One unit (U) of cellulase activity toward CMC was defined as the amount of protein required to release 1 μmol of reducing sugar per min under standard assay conditions, and specific activity was defined as units mg^− 1^ protein.

### Determination of optimal pH and pH stability

The optimal pH of RsEG_m_ was evaluated in 50 mM B & R buffer at 37 °C and pH between 3.0 and 11.0 using 0.5% CMC as the substrates, and all enzymatic reactions under different condition were incubated for 15 min. Specific activities were determined. All enzymatic assays were done in triplicate.

The pH stability assay was estimated by first pre-incubating RsEG_m_ in 50 mM B & R buffer at different pH values (pH 3.0 to 11.0) at 4 °C for 1, 5, 24 and 120 h respectively. The residual activities were then measured immediately under standard conditions (optimal pH, 37°C for 15 min). The initial activity at optimal pH (5.0) was taken as 100%, and the percentage of the residual activity at different time points and pH values against the original one at optimal pH (5.0) was calculated.

### Determination of optimal temperature and thermal stability

The optimal temperature was determined pH 5.0 or 6.0 (50 mM B & R buffer) between 20 and 65°C using CMC (0.5%) as the substrate, and all enzymatic reactions under different condition were incubated for 15 min. Specific activities were determined. All enzymatic assays were carried out in triplicate.

To determine the thermal stability of RsEG_m_, it was pre-incubated for varied time intervals (15 min to 2 h) at pH 5.0, and 30°C, 40°C and 50°C respectively, and samples were chilled on ice for at least 10 min. Afterwards the residual activities were measured under standard conditions (optimal pH, 37°C for 15 min). The experiments at 30°C and 40°C were done with 0.1 mg/ml of RsEG_m_, whereas the ones at 50°C were done with 0.3 mg/ml of RsEG_m_. The original activity at pH 5.0 and 37°C was taken as 100%, and the percentage of the residual activity at different time points and temperatures against the initial one was calculated.

### Determination of kinetic parameters

Kinetic parameters were determined under initial rate conditions using non-linear regression analysis of the Michaelis–Menten equation. Cellulolytic activity was measured at 37°C using CMC as substrate at concentrations ranging from 0.2 to 2% (*w*/*v*) in a 50 mM B & R buffer (pH 5.0). The release of reducing sugars was quantified as above after being incubated for 5 min. All assays were done in triplicate.

### Effects of divalent metal ions on enzyme activity

The stimulatory or inhibitory effects of divalent metal ions (1 mM) on the catalytic activity of RsEG_m_ were determined by adding 1 mM of various divalent metals (Pb(CH_3_COO)_2_, NiSO_4_, MnSO_4_, CuSO_4_, BaCl_2_, ZnSO_4_, CoCl_2_, CaCl_2_, MgCl_2_ and FeSO_4_) to the standard enzyme assay system as above. Since phosphate in B & R buffer might impact the assay, 100 mM sodium acetate (pH 5.0) was used for these assays instead. The system without supplying divalent metal ions was used as the control. The activity of the control at pH 5.0 and 37°C was taken as 100%, and the percentage of the activity in the presence of different divalent metal ions against the control was calculated. All enzymatic assays were done in triplicate.

## Additional files

Additional file 1: Sequence alignment of biochemically characterized insect-origin GHF9 endocellulases. (DOCX 535 kb)

Additional file 2: Codon-optimized gene sequence of RsEG_m_ from *Reticulitermes speratus* with three mutations. (DOCX 14 kb)

Additional file 3: SDS-PAGE analysis of overexpressed pJL36A, pJl36C, pJL36E, pJL36G and pJL36I after induced for 72 h. (DOCX 577 kb)

Additional file 4: SDS-PAGE analysis of overexpressed pJL36A, pJl36C and pJL36E over different time intervals. (DOCX 1251 kb)

Additional file 5: Western blot analysis of overexpressed pJL36C induced for 72 h. (DOCX 249 kb)

## Abbreviations

BGLs: β-glucosidases
CBHs: *Cellobiohydrolases*
CMC: Carboxymethyl Cellulose
EGLs: *Endoglucanases*
GHF: Glycoside hydrolase families

## References

[1] Menon V, Rao M (2012). Trends in bioconversion of lignocellulose: biofuels, platform chemicals & biorefinery concept. Progr Energy Combust Sci.

[2] Hasunuma T, Okazaki F, Okai N, Hara KY, Ishii J, Kondo A (2013). A review of enzymes and microbes for lignocellulosic biorefinery and the possibility of their application to consolidated bioprocessing technology. Bioresour Technol.

[3] Zhang Y-HP, Himmel ME, Mielenz JR (2006). Outlook for cellulase improvement: screening and selection strategies. Biotechnol Adv.

[4] Himmel ME, Xu Q, Luo Y, Ding S-Y, Lamed R, Bayer EA (2010). Microbial enzyme systems for biomass conversion: emerging paradigms. Biofuels.

[5] Henrissat B (1991). A classification of glycosyl hydrolases based on amino acid sequence similarities. Biochem J.

[6] Ohkuma M (2003). Termite symbiotic systems: efficient bio-recycling of lignocellulose. Appl Microbiol Biotechnol.

[7] Ni J, Tokuda G (2013). Lignocellulose-degrading enzymes from termites and their symbiotic microbiota. Biotechnol Adv.

[8] König H, Li L, Fröhlich J (2013). The cellulolytic system of the termite gut. Appl Microbiol Biotechnol.

[9] Lo N, Tokuda G, Watanabe H, Bignell DE, Roisin Y, Lo N (2011). Evolution and function of endogenous termite cellulases. Biology of termites: a modern synthesis.

[10] Brune A (1998). Termite guts: the world’s smallest bioreactors. Trends Biotechnol.

[11] Brune A (2014). Symbiotic digestion of lignocellulose in termite guts. Nat Rev Microbiol.

[12] Nakashima K, Watanabe H, Saitoh H, Tokuda G, Azuma JI (2002). Dual cellulose-digesting system of the wood-feeding termite, *Coptotermes formosanus* Shiraki. Insect Biochem Mol Biol.

[13] Watanabe H, Tokuda G (2010). Cellulolytic systems in insects. Annu Rev Entomol.

[14] Watanabe H, Noda H, Tokuda G, Lo N (1998). A cellulase gene of termite origin. Nature.

[15] Tokuda G, Lo N, Watanabe H, Arakawa G, Matsumoto T, Noda H (2004). Major alteration of the expression site of endogenous cellulases in members of an apical termite lineage. Mol Ecol.

[16] Woon JS, King PJH, Mackeen MM, Mahadi NM, Wan Seman WMK, Broughton WJ, Abdul Murad AM, Abu Bakar FD (2017). Cloning, production and characterization of a glycoside hydrolase family 7 enzyme from the gut microbiota of the termite *Coptotermes curvignathus*. Mol Biotechnol.

[17] Warnecke F, Luginbuhl P, Ivanova N, Ghassemian M, Richardson TH, Stege JT (2007). Metagenomic and functional analysis of hindgut microbiota of a wood-feeding higher termite. Nature.

[18] Burnum KE, Callister SJ, Nicora CD, Purvine SO, Hugenholtz P, Warnecke F, Scheffrahn RH, Smith RD, Lipton MS (2011). Proteome insights into the symbiotic relationship between a captive colony of *Nasutitermes corniger* and its hindgut microbiome. ISME J.

[19] Tokuda G, Lo N, Watanabe H, Slaytor M, Matsumoto T, Noda H (1999). Metazoan cellulase genes from termites: intron/exon structures and sites of expression. Biochim Biophys Acta.

[20] Kim N, Choo YM, Lee KS, Hong SJ, Seol KY, Je YH, Sohn HD, Jin BR (2008). Molecular cloning and characterization of a glycosyl hydrolase family 9 cellulase distributed throughout the digestive tract of the cricket *Teleogryllus emma*. Comp Biochem Physiol B Biochem Mol Biol.

[21] Zhang D, Lax AR, Raina AK, Bland JM (2009). Differential cellulolytic activity of native-form and C-terminal tagged-form cellulase derived from *Coptotermes formosanus* and expressed in *E. coli*. Insect Biochem Mol Biol.

[22] Zhang DH, Lax AR, Bland JM, Allen AB (2011). Characterization of a new endogenous endo-β-1,4-glucanase of Formosan subterranean termite (*Coptotermes formosanus*). Insect Biochem Mol Biol.

[23] Zhou X, Kovaleva ES, Wu-Scharf D, Campbell JH, Buchman GW, Boucias DG, Scharf ME (2010). Production and characterization of a recombinant β-1,4-endoglucanase (glycohydrolase family 9) from the termite *Reticulitermes flavipes*. Arch Insect Biochem Physiol.

[24] Willis JD, Oppert B, Oppert C, Klingeman WE, Jurat-Fuentes JL (2011). Identification, cloning, and expression of a GHF9 cellulase from *Tribolium castaneum* (Coleoptera: Tenebrionidae). J Insect Physiol.

[25] Shirley D, Oppert C, Reynolds TB, Miracle B, Oppert B, Klingeman WE, Jurat-Fuentes JL (2014). Expression of an endoglucanase from *Tribolium castaneum* (TcEG1) in *Saccharomyces cerevisiae*. Insect Sci.

[26] Cairo JP, Oliveira LC, Uchima CA, Alvarez TM, Citadini AP, Cota J, Leonardo FC, Costa-Leonardo AM, Carazzolle MF, Costa FF, Pereira GA, Squina FM (2013). Deciphering the synergism of endogenous glycoside hydrolase families 1 and 9 from *Coptotermes gestroi*. Insect Biochem Mol Biol.

[27] Ni J, Wu Y, Yun C, Yu M, Shen Y (2014). cDNA cloning and heterologous expression of an endo-β-1,4-glucanase from the fungus-growing termite *Macrotermes barneyi*. Arch Insect Biochem Physiol.

[28] Ni J, Takehara M, Watanabe H (2005). Heterologous overexpression of a mutant termite cellulase gene in *Escherichia coli* by DNA shuffling of four orthologous parental cDNAs. Biosci Biotechnol Biochem.

[29] Ni J, Takehara M, Watanabe H (2010). Identification of activity related amino acid mutations of a GH9 termite cellulase. Bioresour Technol.

[30] Hirayama K, Watanabe H, Tokuda G, Kitamoto K, Arioka M (2010). Purification and characterization of termite endogenous β-1,4-endoglucanases produced in *Aspergillus oryzae*. Biosci Biotechnol Biochem.

[31] Todaka N, Nakamura R, Moriya S, Ohkuma M, Kudo T, Takahashi H, Ishida N (2011). Screening of optimal cellulases from symbiotic protists of termites through expression in the secretory pathway of *Saccharomyces cerevisiae*. Biosci Biotechnol Biochem.

[32] Sasaguri S, Maruyama J, Moriya S, Kudo T, Kitamoto K, Arioka M (2008). Codon optimization prevents premature polyadenylation of heterologously-expressed cellulases from termite-gut symbionts in *Aspergillus oryzae*. J Gen Appl Microbiol.

[33] Daly R, Hearn MT (2005). Expression of heterologous proteins in *Pichia pastoris*: a useful experimental tool in protein engineering and production. J Mol Recognit.

[34] Valencia JA, Wang H, Siegfried BD (2014). Expression and characterization of a recombinant endoglucanase from western corn rootworm, in *Pichia pastoris*. J Insect Sci.

[35] Kasana RC, Gulati A (2011). Cellulases from psychrophilic microorganisms: a review. J Basic Microbiol.

[36] Sambrook J, Fritsch EF, Maniatis T (1989). Molecular cloning a laboratory manual.

[37] Miller GL (1959). Use of dinitrosalicylic acid for determination of reducing sugar. Anal Chem.

