# Supplementary material for: Heterologous expression and biochemical characterization of a GHF9 endoglucanase from the termite Reticulitermes speratus in Pichia pastoris
Source: BMC Biotechnol. 2018 Jun 1;18:35. doi: 10.1186/s12896-018-0432-3 (PMC5984754; doi:10.1186/s12896-018-0432-3)

**Additional file 1 – Sequence alignment of biochemically characterized insect-origin GHF9 endocellulases.**

RsEG, CfEG3a, CfEG5a, Cell-1, NtEG, MbEG1, and TcEG1 are from *Reticulitermes speratus*, *Coptotermes formosanus*, *Coptotermes formosanus*, *Reticulitermes flavipes*, *Nasutitermes takasagoensis*, *Macrotermes barneyi*, and *Teleogryllus emma* respectively. The accession numbers of RsEG, CfEG3a, CfEG5a, Cell-1, NtEG, MbEG1, and TeEG-I in GenBank are BAA31326.1, ACI45756.1, ADB12483.1, [AAU20853.2](https://www.ncbi.nlm.nih.gov/protein/71904973?report=genbank&log$=prottop&blast_rank=2&RID=P6RGVKJW014), [BAA33708.1](https://www.ncbi.nlm.nih.gov/protein/3721826?report=genbank&log$=prottop&blast_rank=17&RID=P6RGVKJW014), [AFD33365.1](https://www.ncbi.nlm.nih.gov/protein/380452610?report=genbank&log$=prottop&blast_rank=13&RID=P6RGVKJW014) and ABV32557.1 separately.


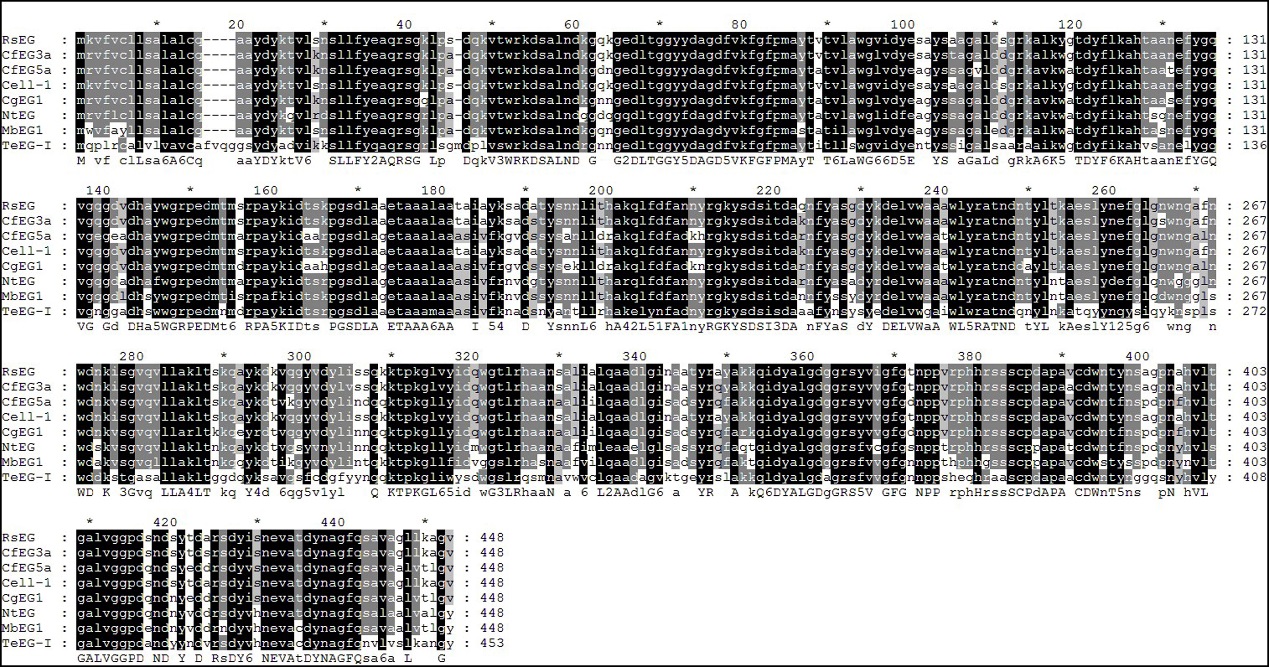

Supplement: Supplementary file 1 — Sequence alignment of biochemically characterized insect-origin GHF9 endocellulases. (DOCX 535 kb) [file 12896_2018_432_MOESM1_ESM.docx]
