# Supplementary material for: Heterologous expression and biochemical characterization of a GHF9 endoglucanase from the termite Reticulitermes speratus in Pichia pastoris
Source: BMC Biotechnol. 2018 Jun 1;18:35. doi: 10.1186/s12896-018-0432-3 (PMC5984754; doi:10.1186/s12896-018-0432-3)
Supplement: Supplementary file 2 — Codon-optimized gene sequence of RsEGm from Reticulitermes speratus with three mutations. (DOCX 14 kb) [file 12896_2018_432_MOESM2_ESM.docx]

**Additional file 2 – Codon-optimized gene sequence of RsEG_m_ from *Reticulitermes speratus* with three mutations**

GCTTACGACTACAAGACGGTTCTTTCCAACTCCCTACTTTTCTACGAGGCTCAACGAAGTGGAAAGTTGCCTAGTGACCAAAAGGTAACGTGGAGAAAAGACTCTGCTCTTAACGACAAGGGTCAAAAGGGTGAAGACCTAACAGGAGGATACTACGATGCTGGTGACTTTGTAAAGTTCGGTTTCCCTATGGCTTACACAGTAACAGTACTGGCTTGGGGTGTTATTGACTACGAGTCAGCTTACTCTGCAGCAGGAGCTTTAGATTCTGCTAGAAAGGCTTTAAAATGGGGTACGGACTACTTCCTGAAGGCTCATACAGCTGCTAATGAGTTCTACGGACAAGTTGGACAAGGAGATGTTGACCATGCATACTGGGGAAGACCAGAAGATATGACTATGAGTAGACCTGCATACAAAATCGACACCTCCAAACCAGGTTCTGACCTTGCAGCAGAAACTGCAGCTGCTCTTGCAGCTACTGCAATCGCATATAAGTCAGCAGATGCAACTTATTCAAACAACCTGATAACACACGCAAAGCAACTGTTCGACTTTGCAAACAACTACCGTGGAAAATACTCCGACTCCATCACTGACGCTCAAAACTTTTACGCCTCCGGCGATTATAAGGATGAGCTAGTTTGGGCTGCAGCATGGTTGTATAGAGCTACCAATGATAATACCTACCTGACTAAGGCTGAATCACTATATAACGAATTTGGGTTGGGCAACTGGAACGGTGCCTTTAACTGGGATAACAAGATTTCCGGTGTCCAGGTCCTTCTGGCCAAATTGACTTCTAAACAGGCCTACAAAGATAAAGTGCAGGGTTACGTCGATTATTTGATTTCCTCTCAGAAGAAAACCCCCAAAGGCTTAGTCTATATCGACCAGTGGGGTACCTTGAGGCACGCCGCCAACTCTGCTTTGATTGCTTTACAGGCTGCTGATTTGGGTATTAATGCCGCTACTTATCGAGCTTATGCCAAGAAACAAATTGATTATGCCTTGGGCGATGGAGGTAGGAGTTATGTGATAGGATTTGGTACTAATCCACCCGTGAGACCTCACCATAGAAGTTCTTCATGCCCTGATGCCCCAGCTGTGTGTGATTGGAATACTTATAATTCTGCCGGGCCAAATGCTCATGTCTTGACCGGAGCTTTGGTTGGTGGTCCAGATTCTAATGATAGTTATACCGATGCCCGTTCTGATTATATTTCAAATGAAGTTGCCACAGATTATAATGCCGGGTTTCAATCAGCCGTTGCCGGCTTATTAGCCGCCGGGGTT
