# Supplementary material for: Heterologous expression and biochemical characterization of a GHF9 endoglucanase from the termite Reticulitermes speratus in Pichia pastoris
Source: BMC Biotechnol. 2018 Jun 1;18:35. doi: 10.1186/s12896-018-0432-3 (PMC5984754; doi:10.1186/s12896-018-0432-3)

**Additional file 3 – SDS-PAGE analysis of overexpressed pJL36A, pJl36C, pJL36E, pJL36G and pJL36I after induced for 72 hr.** Lane 1, Protein marker; Lane 2, pJL36A; Lane 3, pJL36C; Lane 4, pJL36E; Lane 5, pJL36G; Lane 6, pJL36I.


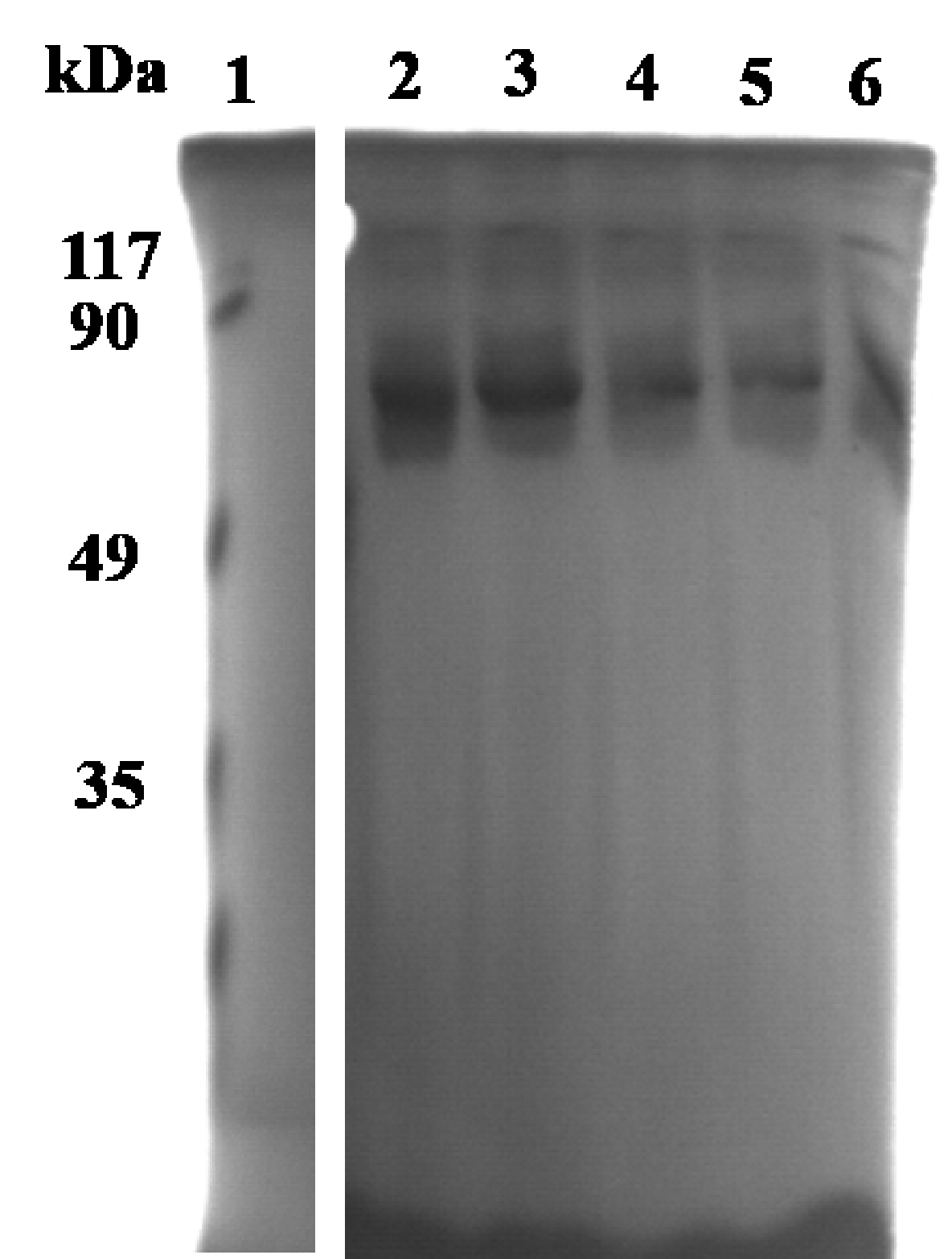

Supplement: Supplementary file 3 — SDS-PAGE analysis of overexpressed pJL36A, pJl36C, pJL36E, pJL36G and pJL36I after induced for 72 h. (DOCX 577 kb) [file 12896_2018_432_MOESM3_ESM.docx]
