# Supplementary material for: Heterologous expression and biochemical characterization of a GHF9 endoglucanase from the termite Reticulitermes speratus in Pichia pastoris
Source: BMC Biotechnol. 2018 Jun 1;18:35. doi: 10.1186/s12896-018-0432-3 (PMC5984754; doi:10.1186/s12896-018-0432-3)

**Additional file 4 – SDS-PAGE analysis of overexpressed pJL36A, pJl36C and pJL36E over different time intervals.** Lane 1, Protein marker; Lanes 2-4, pJL36A was induced for 24 hr, 48 hr and 72 hr respectively; Lanes 5-7, pJL36C was induced for 24 hr, 48 hr and 72 hr respectively; Lanes 8-10, pJL36E was induced for 24 hr, 48 hr and 72 hr respectively.


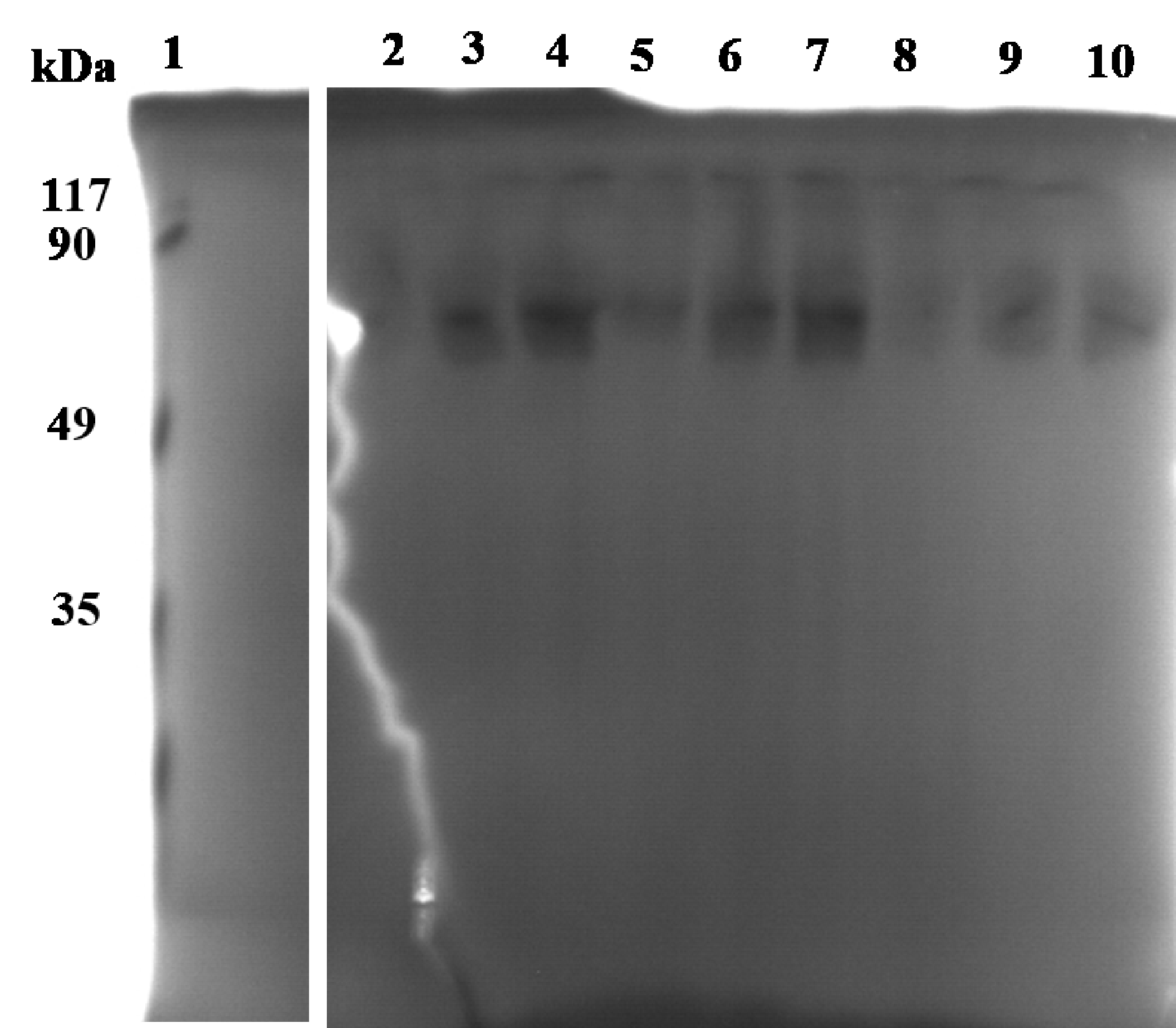

Supplement: Supplementary file 4 — SDS-PAGE analysis of overexpressed pJL36A, pJl36C and pJL36E over different time intervals. (DOCX 1251 kb) [file 12896_2018_432_MOESM4_ESM.docx]
