# Supplementary figures and images for: Heterologous expression and biochemical characterization of a GHF9 endoglucanase from the termite Reticulitermes speratus in Pichia pastoris
Source: BMC Biotechnol. 2018 Jun 1;18:35. doi: 10.1186/s12896-018-0432-3 (PMC5984754; doi:10.1186/s12896-018-0432-3)

**Additional file 5 – Western blot analysis of overexpressed pJL36C induced for 72 hr.**


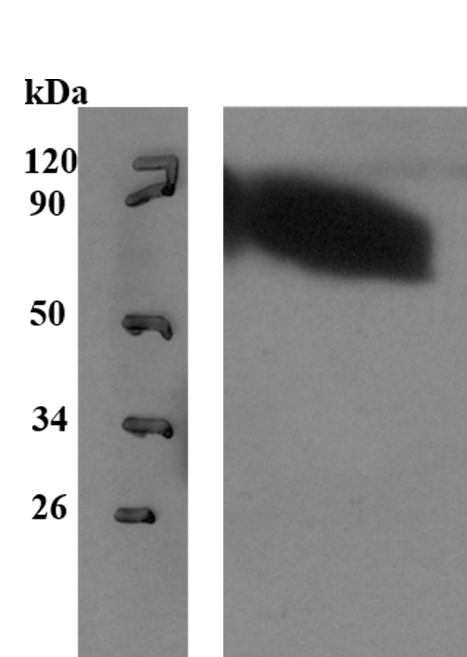

Supplement: Supplementary file 5 — Western blot analysis of overexpressed pJL36C induced for 72 h. (DOCX 249 kb) [file 12896_2018_432_MOESM5_ESM.docx]
